# Association Between Morphological Variations of Mandibular Bone and Pharyngeal Airways: A Morphometric Cone-Beam Computed Tomography (CBCT) Study

**DOI:** 10.7759/cureus.95859

**Published:** 2025-10-31

**Authors:** Gerardo Martínez-Suarez, Luis Pablo Cruz-Hervert, S. Aída Borges-Yáñez, María Eugenia Jiménez-Corona, Martha Elizabeth Tovar-Martínez, Renata Báez-Saldaña, Jean Marc Retrouvey, Viviana Toro-Ibacache

**Affiliations:** 1 Dentistry, Master's and Doctoral Program in Medical, Dental, and Health Sciences, Universidad Nacional Autónoma de México, Mexico City, MEX; 2 Dentistry, Universidad Nacional Autónoma de México, Mexico City, MEX; 3 Epidemiology, Instituto Nacional de Cardiología Ignacio Chávez, Mexico City, MEX; 4 Dentistry, Universidad Nacional Autónoma de México, Tlalnepantla de Baz, MEX; 5 Education, Instituto Nacional de Enfermedades Respiratorias, Universidad Nacional Autónoma de México, Mexico City, MEX; 6 Molecular Genetics, Baylor College of Medicine, Dallas, USA; 7 Dentistry, Instituto de Investigación en Ciencias Odontológicas, Universidad de Chile, Santiago, CHL

**Keywords:** 3d imaging in orthodontics, craniofacial orthodontics, dental morphometrics, mandibular shape, pharyngeal airway, pharyngeal shape, positional obstructive sleep apnea, skeletal classification, skeletal malocclusion, upper airway space

## Abstract

The 3D relationship between mandibular morphology and the upper pharyngeal airway (UPA) configuration remains unclear. Precise anatomical classification can improve risk stratification for obstructive sleep apnea (OSA) and guide orthodontic or surgical interventions.

Objective: To identify the association between form variations of the mandibular bone and pharyngeal airways using cone-beam computed tomography (CBCT).

Methods: This cross-sectional study included 282 CBCT records (aged 9-50 years). Thirty-two mandibular and 20 UPA landmarks were digitized in 3D. Raw landmark coordinates were analyzed without Procrustes adjustment and re-centered at mandibular landmark 2 to preserve the size and spatial position. Exploratory factor analysis (EFA) with VARIMAX rotation was performed, and three latent dimensions were retained for each structure. Based on these factors, hierarchical cluster analysis identified three mandibular and three UPA phenotypes. Multinomial logistic regression adjusted for age, sex, and skeletal class was used to quantify the associations between the mandibular and airway groups.

Results: Mandibular factors explained 95.7% of the morphological variance, and UPA factors explained 99.3%. Md-G1 and Md-G2 exhibited vertical growth with narrow basal widths, whereas Md-G3 showed a broad horizontal pattern. The UPA groups differed mainly at the oropharyngeal level: UPA1 displayed mid-level transverse constriction, UPA2 showed generalized anteroposterior narrowing, and UPA3 maintained a wide and nearly circular lumen. Compared to Md-G3, Md-G1 was associated with a higher probability of UPA1 (relative risk ratio (RRR)=2.60; 95% CI: 0.94-7.23; p=0.066) and UPA2 (RR=2.96; 95% CI: 1.18-7.45; p=0.021). Md-G2 also correlated with UPA1 (RR=2.81; 95% CI: 1.02-7.78; p=0.045). Age contributed to an increased likelihood of UPA2 in older groups, while skeletal class II showed no significant association with UPA2 (RR=1.83; p=0.214). Sex differences were not statistically significant.

Conclusions: 3D morphometric and cluster analyses suggested an association between mandibular and UPA phenotypes. Vertical and narrow mandibular patterns (Md-G1, Md-G2) were linked to airway narrowing (UPA1, UPA2), whereas broad horizontal morphologies (Md-G3) corresponded to a wider airway (UPA3). These findings suggest that mandibular morphology may serve as a structural indicator of airway risk.

## Introduction

The upper pharyngeal airway (UPA) plays a crucial role in respiratory physiology by facilitating the passage of air into the lungs during inspiration. The UPA represents anatomically dynamic and vulnerable segments whose shape characteristics, such as volume, configuration, and minimum area, directly influence airflow and respiratory resistance. A reduction in these dimensions can cause intermittent hypoxia, sleep disturbances, and progressive deterioration of respiratory function [1,2].

Obstructive sleep apnea syndrome (OSAS), initially described by Guilleminault [2], and the dimensions of the UPA are fundamental and widely studied clinical components of this structural and functional alteration. With an estimated prevalence between 5% and 14% in adults [3], OSAS is associated with multiple systemic consequences such as high blood pressure, cardiovascular disease, neurocognitive disorders, and metabolic disorders [3]. This evidence has prompted interest in identifying the anatomical components that predispose individuals to pharyngeal collapse and contribute to the pathogenesis of the disorder.

The craniofacial shape, particularly the structure of the jaw, has been extensively studied in relation to pharyngeal anatomy as an integral part of a multidisciplinary approach to the treatment of OSAS [4-8]. The pharyngeal airway is delimited anteriorly by the maxillomandibular complex and posteriorly by the cervical vertebrae, both of which are easily modified by the mandibular shape and position [1,7,8]. Thus, pharyngeal patency, its functional opening, depends on the interaction between the mandibular shape and soft tissues that constitute the outline of the pharyngeal space [9].

Previous studies have reported associations between skeletal class [5-8], vertical growth patterns [10,11], gonial angle, mandibular plane angle, lingual volume or hyoid position, and significant variations in pharyngeal dimensions. Additionally, these relationships can vary according to sex [4,12], ethnic group [4,9], and other components. However, most of these studies addressed isolated measures from a 2D perspective or analysis without simultaneously adjusting for multiple clinical covariates, which may limit the applicability of the results.

Advances in technology and medical image processing have enabled precise measurement and analysis of the height, width, and cross-sectional area (CSA) of each airway segment, as well as total volume and minimum CSA, to assess their relationship with skeletal patterns [13]. However, consistent evidence of structural associations between mandibular and airway morphology remains limited, especially when accounting for clinical factors such as age, sex, and skeletal classification. These gaps in knowledge hinder the development of reliable predictive diagnostic tools and complicate clinical evaluation of the anatomical risk of pharyngeal obstruction.

While the impact of craniofacial growth on airway dimensions has been studied in children [14], other studies have focused on adults whose bone structure has achieved a stable form [15]. This condition makes it possible to evaluate 3D structural relationships with greater precision without interference from active developmental processes.

Previous research has indicated that a mandible positioned more anteriorly contributes to a broader inferior airway, whereas a mandible with posterior or downward rotation is typically linked to a narrower and more vertically elongated airway space [16]. Other studies have also explored the impact of orthodontic treatment on airway dimensions, particularly in patients with obstructive sleep apnea (OSA) [17]. Understanding how mandibular positioning and morphology affect airway dimensions is necessary before exploring their association using cone-beam computed tomography (CBCT) imaging.

Therefore, the objective of this study was to identify associations between mandibular morphology and pharyngeal airway phenotypes using CBCT. We hypothesize that mandibular shapes characterized by greater horizontal projection and basal width are associated with wider and more stable pharyngeal airway configurations. Clinically, this relationship suggests less susceptibility to collapse. This anatomical-functional approach is expected to generate evidence that strengthens current and potential tools for the diagnosis, prevention, and personalized treatment of respiratory disorders associated with certain craniofacial syndromes.

## Materials and methods

This was a cross-sectional study. The study population consisted of 282 CBCT images of patients aged nine to 50 years who attended the Imaging Department of the Division of Postgraduate Studies and Research, Faculty of Dentistry, National Autonomous University of Mexico, between October 2018 and December 2022.

An anonymized database of CBCT scans performed between 2018 and 2022 and arranged chronologically was created by the university’s Department of Imaging. The study sample was obtained through stratified random sampling across defined age groups (9-15, 16-21, 22-27, 28-33, 34-39, and 40-50 years). Within each stratum, participants were randomly selected using a random number generator (Creations App®), and the corresponding scans were retrieved sequentially from the full list of available records.

Using an online calculator (G*Power) with the a priori sample size module for multiple regression, it was estimated that the minimum required sample size was 256 CBCT scans, considering an anticipated effect of 0.10, statistical power of 0.90, 16 predictors (age, sex, skeletal class, UPA type, and mandibular type), and an alpha of 0.05. Additionally, a 20% increase in sample size was included to account for potential data loss. The inclusion criterion was CBCT scans of patients aged nine to 50 years without orthodontic appliances, craniofacial syndromes, or osteosynthesis plates due to orthognathic surgery. The exclusion criterion was CBCT scans with image distortions that prevented clear identification of anatomical and facial structures.

Data collection

Images were obtained during the previously indicated period following a standardized protocol. Patients were scanned in maximum intercuspation, without swallowing or movement during image capture. To minimize postural variability affecting 3D measurements, cephalic positioning was standardized by aligning the Frankfort horizontal plane parallel to the floor and properly orienting the occlusal plane during acquisition. We used a field of view of 150×150 mm. During CBCT acquisition, patients were occluded at maximum intercuspation, maintained a natural head posture, and avoided swallowing to minimize motion artifacts. The NewTom VGi tomograph (CEFLA SC, Verona, Italy) was configured with an amperage of 3-7 mA for pediatric subjects (nine to 15 years) and 7-15 mA for adults, a kilovoltage of 110 kV, one-time exposure of 18 s, and a rotation angle of 180°. The voxel size was set to 0.3×0.3×0.3 mm, and Digital Imaging and Communications in Medicine (DICOM) volumes were loaded into the 3D Slicer program (http://www.slicer.org).

Each volume was cropped to define a specific region of interest (ROI), within which semi-automatic segmentation of the mandible and upper pharyngeal airway (UPA) was performed using 3D Slicer. A median smoothing filter (0.75 mm) was applied to eliminate minor surface irregularities while preserving the overall contour shape, followed by a Gaussian smoothing filter (1.00 mm) to further refine the segmentation. The resulting segmented surfaces were then exported in stereolithography (STL) format. Landmark digitization was performed using the SlicerMorph extension within 3D Slicer [18,19].

For the mandible, 32 landmarks were placed, four in the midline and 14 on each side, as described in a previous study [20], generating a total of 96 variables (32 points×3 coordinates), as shown in Table 1. For the UPA, 20 landmarks (10 unpaired and 10 paired) were digitized, resulting in 60 variables (20×3). The 20 UPA landmarks were established on the anterior and posterior walls of the upper airway at the intersection between the midsagittal plane and horizontal reference lines drawn based on anatomical landmarks of the mandible. The bicondylar line was drawn between points 5 and 19 (the lateral poles of the mandibular condyles), whereas the condylar neck line was used to locate points 2 and 3. The sigmoid notch line identified points 4 and 19. The deepest points on the anterior border of the mandibular ramus were marked at points 6 and 7. The gonial angle was defined using points 16 and 17. Finally, a line along the inferior border of the mandibular symphysis was established between the anterior (10 and 11) and posterior points (12 and 13).

**Table 1:** Description of the landmarks placed on the mandible and upper airways and their corresponding numbers Columns representing mandibular landmarks refer to anatomical points on the mandible, labeled as Md, and grouped as paired (right and left) or unpaired (midline). Columns representing upper airway landmarks indicate anatomical points in the upper airway, labeled as UA, and are also categorized as paired or unpaired (anterior/posterior midline). *Located in the posterior region of the upper airway.

Right		Middle			Left	
Landmarks paired (14)	Landmarks paired (5)	Landmarks unpaired (4)	Anterior unpaired (5)	Posterior unpaired (5)	Landmarks paired (14)	Landmarks paired (5)
Mandibular	Upper airway	Mandibular	Upper airway		Mandibular	Upper airway
Md-5	UA-2	Md-1	UA-1	UA-20*	Md-5’	UA-3
Md-6	UA-6	Md-2	UA-4	UA-18*	Md-6’	UA-7
Md-7	UA-10	Md-3	UA-5	UA-15*	Md-7’	UA-11
Md-8	UA-12	Md-4	UA-8	UA-14*	Md-8’	UA-13
Md-9	UA-16	-	UA-9	UA-19*	Md-9’	UA-17
Md-10	-	-	-	-	Md-10’	-
Md-11	-	-	-	-	Md-11’	-
Md-12	-	-	-	-	Md-12’	-
Md-13	-	-	-	-	Md-13’	-
Md-14	-	-	-	-	Md-14’	-
Md-15	-	-	-	-	Md-15’	-
Md-16	-	-	-	-	Md-16’	-
Md-17	-	-	-	-	Md-17’	-
Md-18	-	-	-	-	Md-18’	-

The 3D coordinates of the landmarks served as the basis for all analytical procedures. Each landmark was represented by three spatial coordinates: X (lateral-medial axis), Y (superior-inferior axis), and Z (anterior-posterior axis). These coordinates describe the exact geometric location of each point in 3D space, allowing the spatial relationships and anatomical shapes of the structures to be preserved (Table 1).

**Figure 1:**
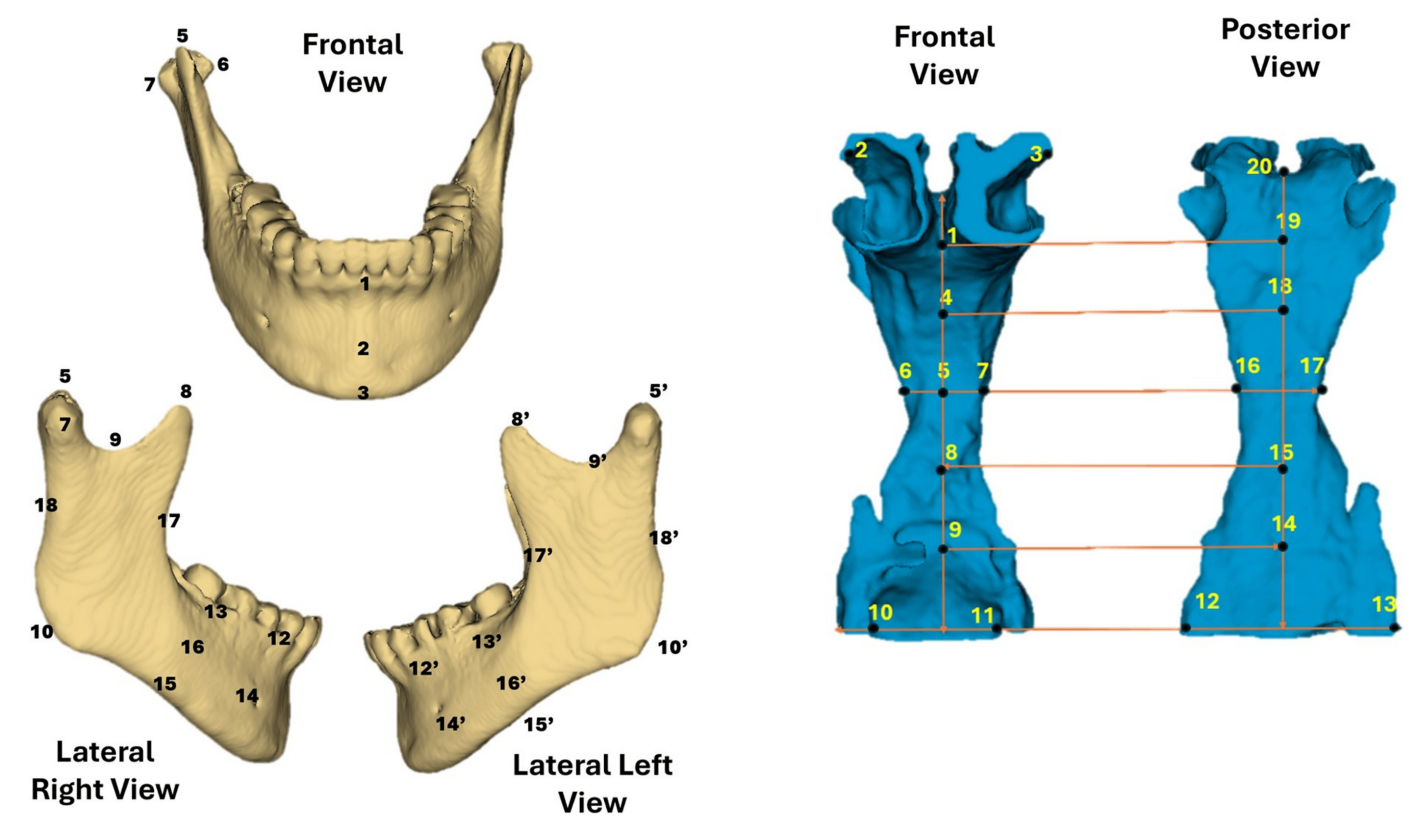
Mandibular and UPA landmarks Mandibular and UPA landmarks: 3D representation (STL) of the mandible (yellow) and UPA (blue) showing the spatial arrangement of the anatomical landmarks used in morphometric analyses. Six anatomical views are presented: frontal, right lateral, left lateral, posterior, superior anterior, and superior posterior. The horizontal lines and dark dots indicate standardized locations using the SlicerMorph extension in 3D Slicer, which was used to capture the 3D shape of both structures. UPA, upper pharyngeal airway; ​​STL, stereolithography Image Credits: Gerardo Martínez-Suárez

The landmark locations are shown in Figure 1. The 3D coordinates of the landmarks were exported in comma-separated values (CSV) format to preserve the X, Y, and Z values of each point. These data were organized in an Excel (Microsoft, Redmond, WA) spreadsheet and subsequently analyzed using the statistical package Stata v15.0 (StataCorp, College Station, TX, USA).

EFA was performed using raw landmark coordinates for the mandible and the upper airway. To avoid translation effects, mandibular landmark two (Figure 1) was fixed as the origin of the coordinate system, and all other coordinates were recalculated relative to this point. This procedure preserved the variability in both size and shape, maintaining the anatomical proportions of the mandible and airway in their original context.

The x, y, and z coordinates were analyzed directly without Procrustes adjustment, following the approach suggested by Kang [21]. An EFA with VARIMAX rotation was then performed, in line with the recommendations of Bookstein for using factor analysis instead of principal component analysis (PCA) for morphometric data [22]. This method allows for the identification of latent dimensions from the intercorrelation of landmarks. Prior to this, a Kaiser-Meyer-Olkin (KMO) test was conducted to evaluate sampling adequacy (mandible and UPA KMO value >0.90). Factor retention was based on two criteria: eigenvalues greater than 1.0 and the identification of the inflection point in the scree plot, known as Cattell’s elbow. In our study, a marked inflection was observed between the third and fifth factors. Factor scores were then computed for each individual, and for simplicity, the retained factors were labeled as PF1-PF3 (principal factor 1-principal factor 3).

Based on the scores obtained from the factors (PF1-PF3), hierarchical cluster analysis was performed using Ward’s method and the Euclidean distance. The results were visualized using dendrograms. The number of clusters was determined using visual criteria derived from the dendrogram; the largest vertical jump between successive fusions was identified, and a horizontal cut was made just above this point. The internal cohesion of each resulting subtree was assessed by analyzing the homogeneity of the linkage heights and the absence of abrupt internal jumps. Additionally, the overall pattern of the dendrogram was expected to resemble a “step” structure, characterized by a few low-level fusions followed by one high-level fusion, rather than a gradual “staircase” pattern. Particular attention was given to the relative size of each subtree, and very small residual branches were reviewed as potential outliers. The horizontal order of leaves was disregarded, with linkage height serving as the sole criterion for group formation.

Once k was set, each individual was assigned to a cluster. To characterize each group, the medians of PF1-PF3 were computed per cluster, and the individuals closest in Euclidean distance to the median vector were selected as representative clusters. These cases were used as the average representation of each group to identify the distinct phenotypic groups. Individuals were grouped according to this variation into three mandibular phenotypes: Md-G1 (horseshoe-shaped), Md-G2 (V-shaped), and Md-G3 (U-shaped); and three airway phenotypes: UPA1 (middle narrow shape), UPA2 (overall narrow shape), and UPA3 (wide shape).

Data reliability

Repeated landmarking and segmentation analyses in 20 randomly selected scans demonstrated excellent measurement consistency across all axes, supporting the use of CBCT, whose diagnostic accuracy for 3D localization has been previously validated [23]. Dahlberg errors ranged from 1.36 (X-axis) to 1.63 (Z-axis), indicating high precision with slightly lower reproducibility in the vertical dimension. Intraclass correlation coefficients (ICC) confirmed this robustness, with individual ICCs between 0.959 and 0.979 and average ICCs above 0.986 (all p<0.001). These results indicate that the 3D landmark data are highly reliable, minimizing measurement noise and supporting the validity of the morphometric analyses.

Variables

Dependent Variable

The dependent variable was categorical and represented the morphological variation in the upper airway. These groups, described in detail in the Results section, reflect distinct morphometric patterns identified through 3D analysis. Briefly, they were categorized into three groups: UPA1 ("middle narrow shape"), UPA2 ("overall narrow shape"), and UPA3 ("wide-shaped").

Independent Variable

The independent variable was also categorical and corresponded to the morphological variation groups of the mandible. As detailed in the Results section, the mandibular forms were classified into three categories: Md-G1 ("horseshoe shape"), Md-G2 ("V-shape"), and Md-G3 ("U-shape").

Overlapping shape comparisons between the groups were performed using 3D superimposition of the mean mandibular surfaces corresponding to the 25th and 75th percentiles of the principal factors (PFs) PF1, PF2, and PF3 (using unmodified coordinates). The SlicerCMF (Cranio-Maxillofacial) registration extension within the 3D Slicer software was used for this purpose. In each case, the mandibular model corresponding to the 25th percentile served as the mean reference model, which was fixed for superimposition of the comparison model.

Covariates

Age group (stratified by range), sex (male and female), and skeletal class (class I, II, and III) were included as covariates to adjust the multivariate models and control for potential confounding factors.

Data analysis

A descriptive analysis of the sample was conducted and categorized by age group, considering sociodemographic and clinical characteristics.

Statistical analysis was designed to characterize the patterns of variation in mandibular shape and UPA, identify structural groupings, and explore associations with age, sex, and skeletal class (class I, II, and III), which were included as covariates to adjust the multivariate models and control for potential confounding factors.

EFA was performed individually for each anatomical structure to reduce the dimensionality of the dataset and extract latent factors that summarized the variation in the 3D shape. PFs were extracted, and the number of factors was determined based on eigenvalues greater than one, complemented by visual inspection of a sedimentation graph, as explained above. The retained factors were orthogonally rotated using the VARIMAX method to facilitate structural interpretation. Using this procedure, three PFs were generated for the mandible and three for the UPA, which were used as summary variables in the subsequent analyses.

To explore possible groupings among individuals using PF1-PF3 of the mandible and upper airway shape, a hierarchical cluster analysis was performed using Ward's method. Euclidean distance was applied for both the mandible and UPA. The grouping was based on three PFs extracted from each case. The hierarchical structure was represented by dendrograms, various cutting solutions were explored, and the partition into three groups was selected as the most stable and representative.

Descriptive statistics of the factors in each cluster were calculated to compare shape characteristics among the groups, including mean, standard deviation, and median.

Finally, to test the hypothesis that mandibular morphology characterized by greater anterior projection and anatomical features favoring a broader configuration is associated with a more spacious pharyngeal airway, a multinomial logistic regression model was performed to examine the association between UPA shape and sex, age group, and skeletal class. The mandibular shape group, sex, age group, and skeletal class were included as predictors, and the third group was used as the reference category. The results of the model are expressed as relative risk ratios (RRRs) with a statistical significance level of 5%.

## Results

The details are presented in Table 2. A total of 282 CBCT scans were analyzed, with 170 (60%) female patients and 112 (40%) male patients. Regarding age distribution, 51 (18.1%) scans corresponded to the nine-to-15-year group and 52 (18.4%) to the 16-21-year group. The 22-27-, 28-33-, 34-39-, and 40-50-year groups represented 56 (19.9%), 41 (14.5%), and 31 (10.9%) of the records, respectively. No statistically significant differences were observed between sex distributions across age groups or between sexes (p=0.194).

**Table 2:** Characteristics of the study sample

Age group							
Characteristics	Group 1, 51 (18.09%)	Group 2, 51 (18.09%)	Group 3, 52 (18.44%)	Group 4, 56 (19.86%)	Group 5, 41 (14.54%)	Group 6, 31 (10.99%)	Total, 282 (100%)
Range of age	9-15	16-21	22-27	28-33	34-39	40-50	9-50
Female (freq) (%)	26 (50.98%)	31 (60.78%)	35 (67.31%)	30 (53.57%)	24 (58.54%)	24 (77.42%)	170 (60.28%)
Male (freq) (%)	25 (49.02%)	20 (39.22%)	17 (32.69%)	26 (46.43%)	17 (41.46%)	7 (22.58%)	112 (39.72%)
Age (mean, SD)	(11.5±1.4)	(17.5±1.5)	(23.6±1.5)	(30.5±1.8)	(36.1±1.9)	(45.4±4.1)	(26.0±10.5)
Skeletal classification							
Class I (freq) (%)	15 (29.41%)	17 (33.33%)	19 (36.54%)	18 (32.14%)	15 (36.59%)	8 (25.81%)	92 (32.62%)
Class II (freq) (%)	30 (58.82%)	20 (39.22%)	22 (42.31%)	30 (53.57%)	17 (41.46%)	19 (61.29%)	138 (48.94%)
Class III (freq) (%)	6 (11.76%)	14 (27.45%)	11 (21.15%)	8 (14.29%)	9 (21.95%)	4 (12.90%)	52 (18.44%)

Regarding skeletal classification, 92 (32.6%) of the cases were classified as class I, 136 (48.3%) as class II, and 54 (19.0%) as class III. Similarly, no significant differences were observed in the distribution of skeletal classes across age groups (p=0.412).

Mandibular bone characteristics and group classification

**Figure 2:**
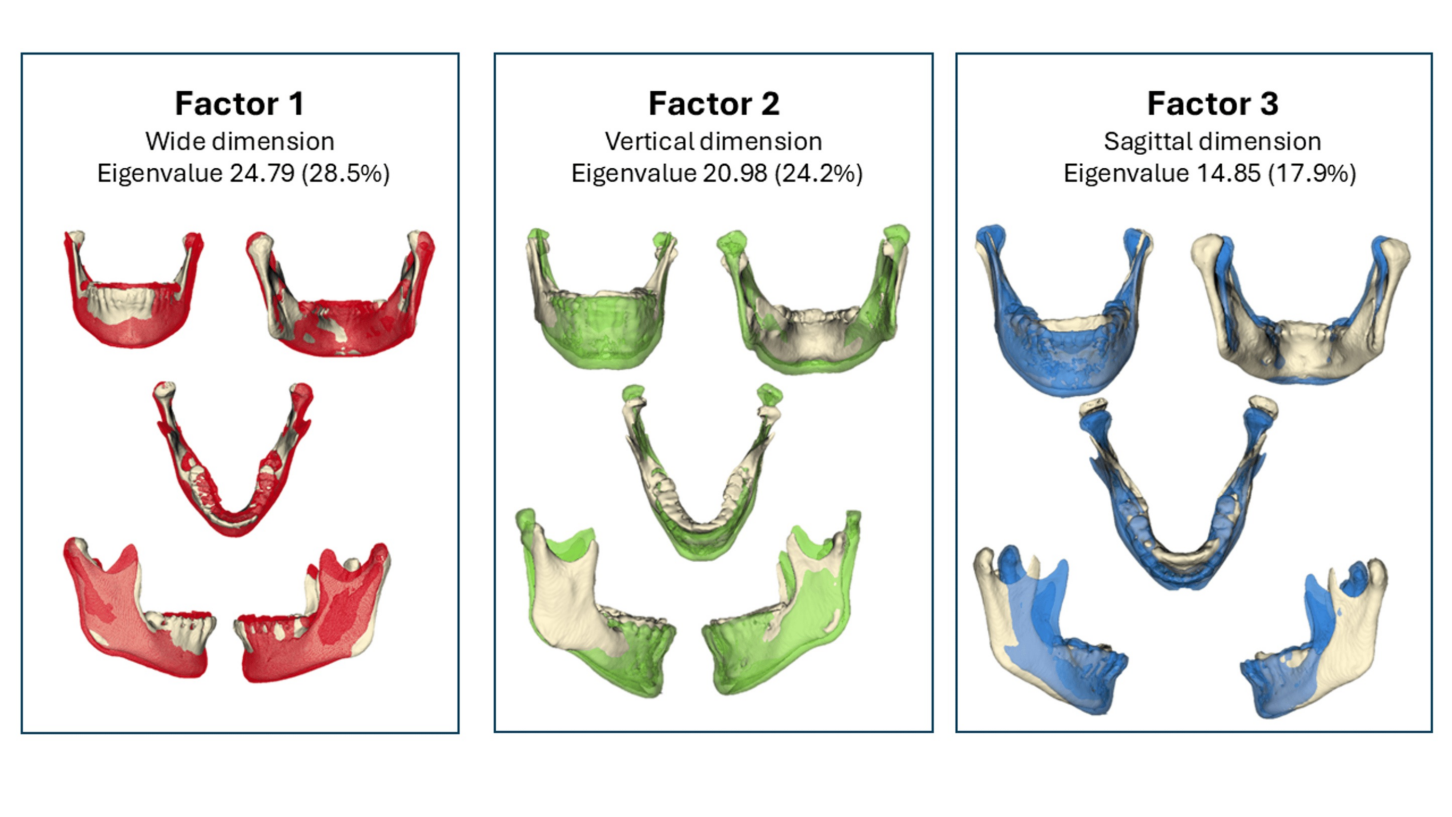
PFs of mandibular variation derived from PCA of 3D landmark coordinates Each figure shows a major factor of shape variability. The colored mandible represents an average model used as the fixed reference, whereas the semitransparent overlays illustrate the shape variation along this PF. Factor 1 represented changes in vertical (height) dimensions, factor 2 corresponded to transverse (width) variations, and factor 3 captured sagittal (anteroposterior) differences. Eigenvalues and percentages of the variance explained by each factor are indicated. The models reflect hypothetical extremes along the axis of each factor to visually demonstrate morphological variation. PF, principal factor; PCA, principal component analysis Image Credits: Gerardo Martínez-Suárez and Luis Pablo Cruz-Hervert

EFA revealed three PFs. PF1 represented a generalized enlargement of the mandible, reflecting increased width and height across all dimensions (i.e., PF1: “wide dimension”). PF2 was characterized by an increase in the gonial angle, with both the mandibular body and ramus displaying growth in a clockwise rotational pattern (i.e., PF2: “vertical dimension”). PF3 was mainly associated with a more anterior positioning of the mandible in the sagittal plane rather than an actual increase in anteroposterior size (i.e., PF3: “sagittal positioning”). Together, these three factors explained more than 70% of the morphological variance (Figure 2).

For each mandible in the sample, standardized scores derived from the factorial analysis were used to classify the specimens into three distinct morphological clusters. These groups showed consistent differences in ramus proportion, mandibular body development, gonial angle, and basal shape.

Group Md-G1 “horseshoe shape” included 123 (43.6%). This cluster represents a well-proportioned mandible with predominantly horizontal development. The ramus is wide, and the mandibular body exhibits a robust transverse growth pattern. The inferior border of the mandibular base adopts a horseshoe configuration, reflecting a balanced morphology with greater horizontal than vertical expression.

Group Md-G2 “V-shape” included 83 (29.4%). This phenotype is characterized by a ramus that is broader in the anteroposterior direction, whereas the mandibular body shows a relatively lower alveolar height. The inferior border of the mandible presents a V-shaped base, indicating a more angular basal pattern.

Group Md-G3 “U-shape” included 76 (27.0%). This group was distinguished by a ramus that was slender in the anteroposterior plane and a narrower mandibular body. The basal configuration is U-shaped, accompanied by a more open gonial angle, indicating a stronger vertical growth tendency.

**Figure 3:**
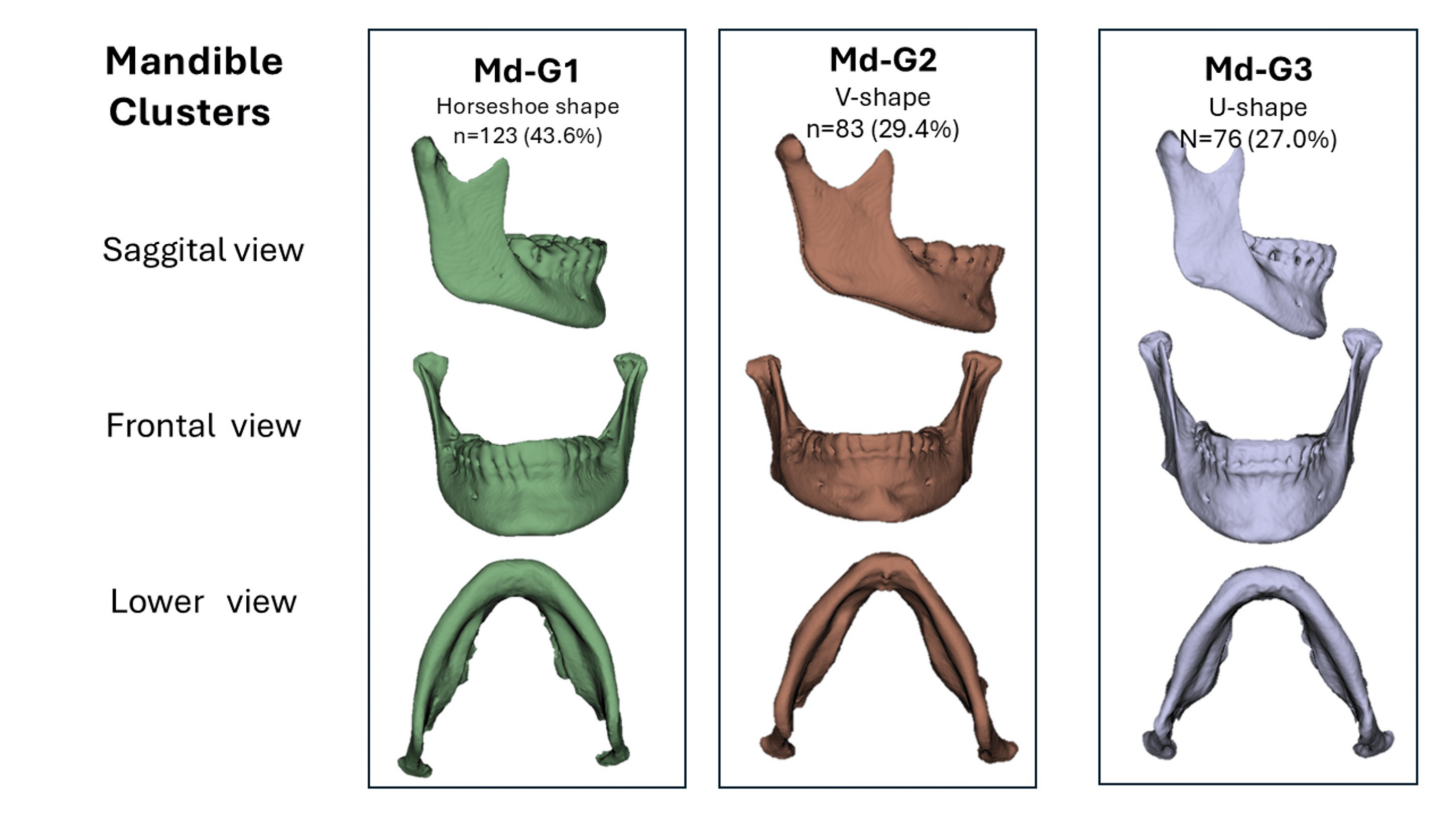
Upper airway morphological clusters based on 3D shape analysis Three groups were identified through hierarchical clustering (Ward's method) based on the PF scores derived from landmark-based shape data. Each group is represented by the average anatomical configuration visualized in the frontal and inferior views. The clustering highlights distinct structural patterns in airway volume and geometry. PF, principal factor Image Credits: Gerardo Martínez-Suárez and Luis Pablo Cruz-Hervert

Owing to their distinctive features, proportions of the ramus, body development, gonial angle, and basal outline, these clusters were designated as “horseshoe shape” (Md-G1), “V-shape” (Md-G2), and “U-shape” (Md-G3), as shown in Figure 3.

Upper pharyngeal airway characteristics and group classification

Overlapping shape comparisons between groups were performed using 3D superimposition of the mean airway surfaces corresponding to the 25th and 75th percentiles of the principal factors (PF1 and PF2) with unmodified landmark coordinates. The SlicerCMF (Cranio-Maxillofacial) registration extension within the 3D Slicer software was used for this purpose. In each case, the UPA model corresponding to the 25th percentile served as the fixed reference model for the superimposition.

**Figure 4:**
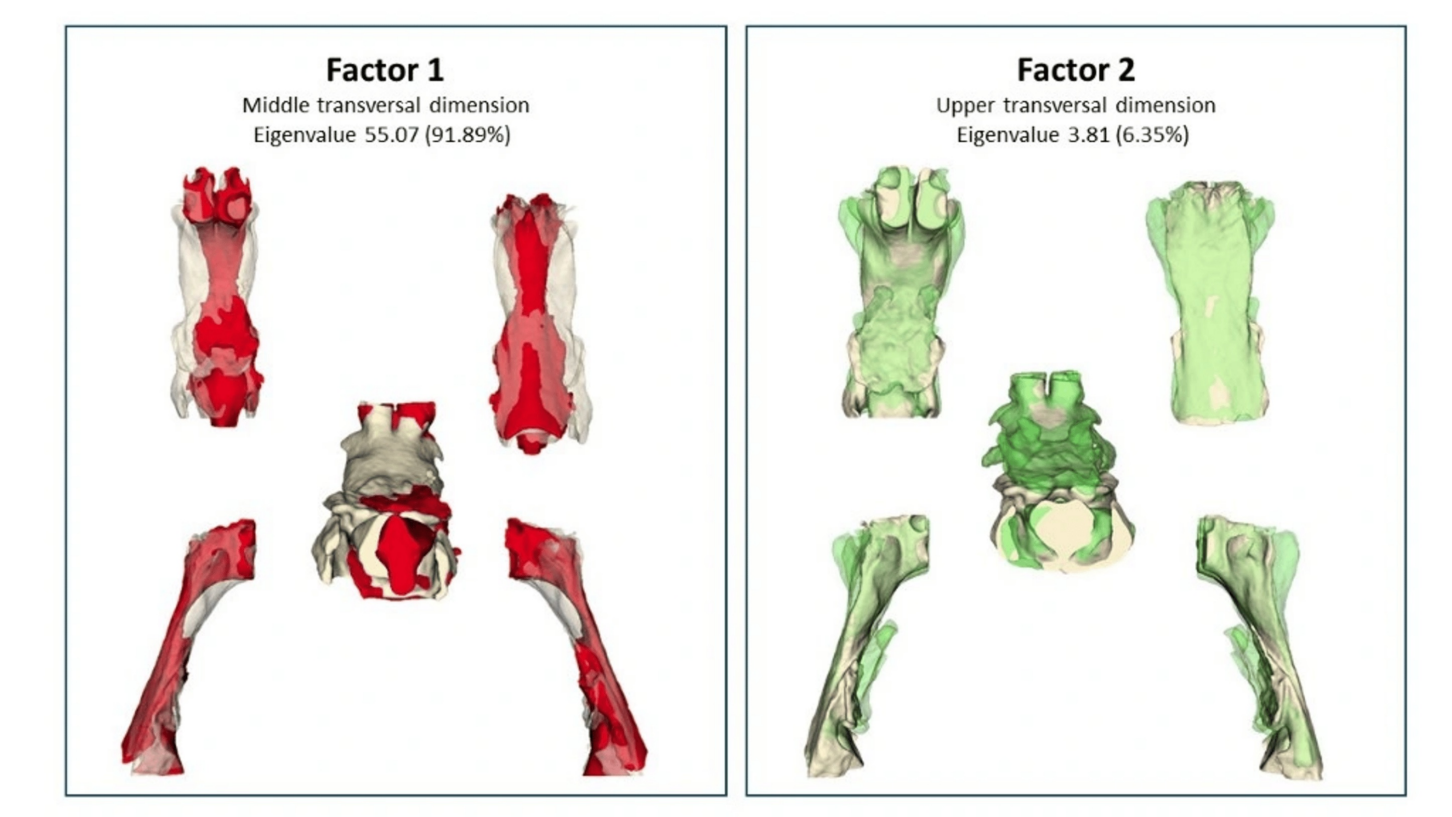
PFs of upper airway form variation PFs of upper airway morphology derived from exploratory factor analysis of 3D landmark coordinates. The three panels illustrate the first three shape factors using superimposition models shown in multiple anatomical views (frontal, posterior, inferior, right, and left). The colored UPA represents the average model used as the fixed reference, while the semitransparent overlays depict the shape variation along each PF. Eigenvalues and percentages of explained variance are indicated for each factor. The color-coded deformations highlight the extremes along each shape axis to visualize morphometric variability. PF, principal factor; UPA, upper pharyngeal airway Image Credits: Gerardo Martínez-Suárez and Luis Pablo Cruz-Hervert

For the UPA, 20 anatomical landmarks were analyzed, and exploratory factor analysis revealed two main factors that together explained 98.2% of the morphological variability. PF1 represented the middle transverse dimension (eigenvalue 55.07; 91.9%), while PF2 captured the upper transverse dimension (eigenvalue 3.81; 6.3%) (Figure 4).

**Figure 5:**
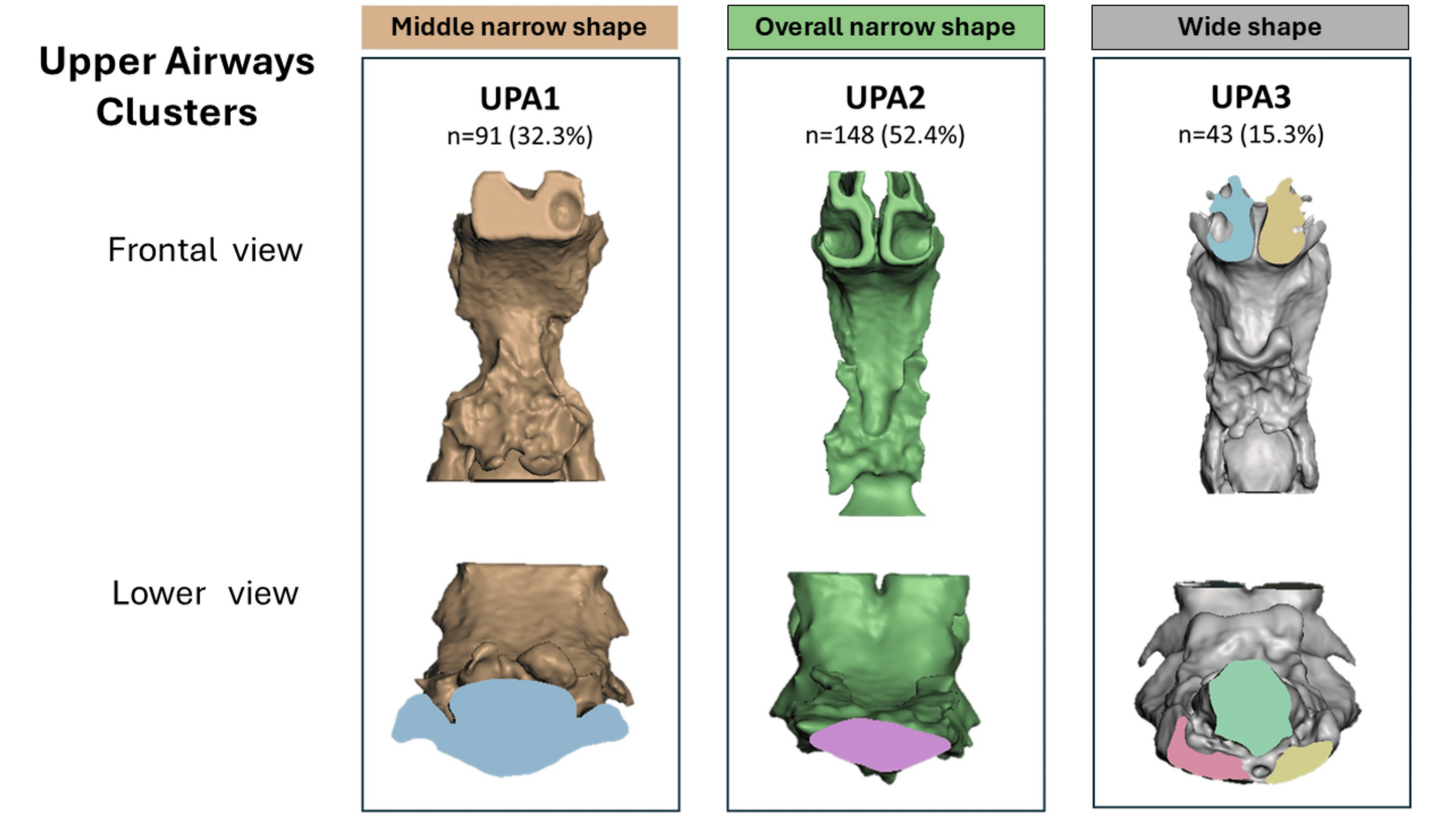
Upper airway morphological clusters based on 3D shape analysis Sankey diagram illustrating the morphological correspondence between mandibular shape clusters (Md-G1, Md-G2, and Md-G3) and upper airway shape clusters (UPA1, UPA2, and UPA3). Image Credits: Gerardo Martínez-Suárez and Luis Pablo Cruz-Hervert

Following the same strategy used for the mandibular complex, standardized scores of the three identified morphometric vectors were calculated for each upper airway: mean transverse width, sagittal depth, and upper-lower vertical continuity. Using these values, a hierarchical cluster analysis was performed, revealing three pharyngeal airway phenotypes (Figure 5).

UPA1 “middle narrow shape” included 91 (32.3%). This phenotype is characterized by a localized constriction in the middle third of the airway, while the upper and lower thirds remain relatively well preserved. The resulting configuration reflects a bottleneck pattern with adequate development at the upper and lower extremes.

UPA2 “overall narrow shape” included 148 (52.4%). This cluster shows a more generalized reduction in airway dimensions across all levels, with the lower third presenting the narrowest configuration. The morphology reflects a uniformly restricted lumen.

UPA3 “wide shape” included 43 (15.3%). This group presented a broader airway, although it was not excessively enlarged. The lower third tended to display a more circular and symmetric contour, in contrast to the oval shapes observed in clusters 1 and 2. This configuration confers greater uniformity to the airway lumen and a low probability of obstructive collapse.

Relationships between groups of changes in the shape of the mandibular and pharyngeal airways

Analysis of categorical variables between mandibular morphology and upper airway (UPA) shape showed a differential distribution among the groups. A statistically significant association was identified (chi-squared test, p=0.037), indicating that specific mandibular configurations tended to correspond to particular airway patterns.

For the Md-G1 (V-shape) group, the most frequent correspondence was with UPA2 (overall narrow shape) in 47 (56.6%) cases, followed by UPA1 (middle narrow shape) in 25 (30.1%) cases and UPA3 (wide shape) in 11 (13.3%) cases. In the Md-G2 (horseshoe shape) cluster, the predominant association was also with UPA2 (overall narrow shape) in 60 (48.8%) cases, while UPA1 (middle narrow shape) accounted for 49 (39.8%) cases and UPA3 (wide shape) for 14 (11.4%) cases. Finally, for the Md-G3 (U-shape) group, the most common pattern was UPA2 (overall narrow shape) in 41 (54.0%) cases, followed by UPA3 (wide shape) in 18 (23.7%) cases and UPA1 (middle narrow shape) in 17 (22.4%) cases.

**Figure 6:**
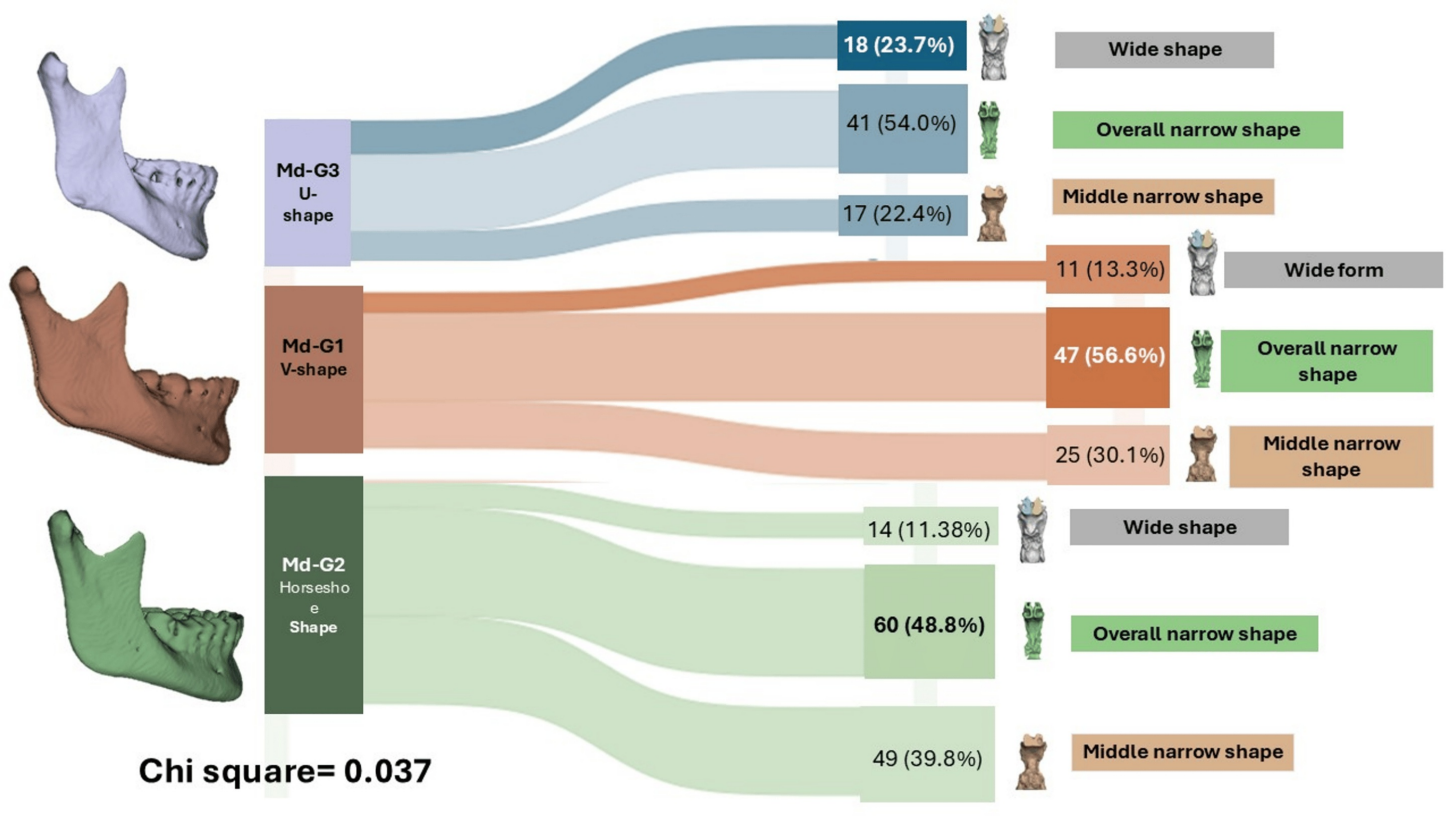
3D morphometric clusters of the upper airway in relation to mandibular shape groups Sankey diagram showing the morphological correspondence between mandibular shape clusters (Md-G1, Md-G2, and Md-G3) and upper airway shape clusters (UPA1, UPA2, and UPA3). Chi-square test result: p=0.037. Image Credits: Gerardo Martínez-Suárez and Luis Pablo Cruz-Hervert

These findings confirm that mandibular configuration influences the variability of airway morphology, reinforcing the correspondence between craniofacial skeletal form and pharyngeal airway phenotype (Figure 6).

Results of the multivariate analysis of the shape of the airways and mandibular shape

In the multinomial logistic regression model, the UPA3 or wide form was established as the reference category corresponding to the configuration of the least frequent form within the sample. This choice implies that all relative risks (RRs) estimated for UPA1 and UPA2 are interpreted in relation to the probability of belonging to UPA3. In other words, each coefficient indicates how many times the probability of belonging to a specific group (one or two) increases (RR>1) or decreases (RR<1) compared to UPA3, according to the value of the variable. Correspondingly, the other variables in the model were kept constant. This approach facilitates comparison between the predominant shape configurations (UPA1 and UPA2) and the contrasting structural shape represented by UPA3 (Table 2).

The multinomial logistic regression model revealed statistically significant associations between mandibular shape patterns and different UPA shape groups, even after adjusting for sex, age, and skeletal class. The reference category for the dependent variable was UPA3 (Table 3).

**Table 3:** Multinomial logistic regression model predicting upper airway morphological groups on clinical variables p-value for Wald test. RRR, relative risk ratio

Pharyngeal upper airways groups	Variable	RRR	95% CI	p-value
Middle narrow shape (UPA1)	Md-G1	2.60	0.94 to 7.23	0.066
	Md-G2	2.81	1.02 to 7.78	0.045
	Md-G3	Reference category		
	Sex			
	Male	2.16	0.94 to 4.99	0.071
	Female	Reference category		
	Age group			
	9 to 15	Reference category		
	15 to 20	0.59	[0.18 to 1.98]	0.396
	20 to 29	0.24	0.06 to 0.90	0.035
	30 to 34	0.29	0.08 to 1.03	0.056
	35 to 39	1.16	0.20 to 6.83	0.862
	40 to 50	0.22	0.05 to 1.08	0.062
	Skeletal class			
	Class III	Reference category		
	Class I	1.06	0.37 to 3.01	0.914
	Class II	0.91	0.33 to 2.51	0.854
Overall narrow shape (UPA2)	Md-G1	2.96	1.18 to 7.45	0.021
	Md-G2	2.09	0.82 to 5.31	0.12
	Md-G3	Reference category		
	Sex			
	Males	1.66	0.74 to 3.66	0.212
	Females	Reference category		
	Age group			
	9 to 15	Reference category		
	15 to 20	2.92	0.75 to 11.37	0.121
	20 to 29	5.15	1.26 to 21.09	0.023
	30 to 34	4.95	1.25 to 19.56	0.022
	35 to 39	24.19	4.12 to 141.74	<0.001
	40 to 50	5.7	1.26 to 26.29	0.024
	Skeletal class			
	Class III	Reference category		
	Class I	0.71	0.26 to 1.95	0.509
	Class II	1.83	0.70 to 4.74	0.214
Wide shape (UPA3)	Reference category			

The results indicated that individuals with an Md-G2 (horseshoe shape) mandible had a significantly higher probability of presenting a UPA1 (middle narrow shape) airway (RR=2.81; 95% CI: 1.02-7.78; p=0.045) than those with Md-G3 (reference). Similarly, Md-G1 (V-shape) showed a strong tendency toward UPA1 (RR=2.60; 95% CI: 0.94-7.23; p=0.066), although this did not reach statistical significance. For UPA2 (overall narrow shape), Md-G1 was significantly associated (RR=2.96; 95% CI: 1.18-7.45; p=0.021), while Md-G3 served as the reference. These findings suggest that both Md-G1 and Md-G2 configurations are more prone to narrower airway morphologies, whereas Md-G3 (U-shape) appears less associated with restriction.

Regarding sex, males tended to show higher probabilities of belonging to UPA1 (RR=2.16; 95% CI: 0.94-4.99; p=0.071) and UPA2 (RR=1.66; 95% CI: 0.74-3.66; p=0.212) than females, though without statistical significance.

Age showed relevant associations: individuals aged 20-29 years were less likely to belong to UPA1 (RR=0.24; 95% CI: 0.06-0.90; p=0.035) compared with the nine- to 15-year group. Conversely, for UPA2, age groups 20-29, 30-34, 35-39, and 40-50 years all showed increased risks (RRs between 4.95 and 24.19; several p<0.05), highlighting a strong age-related tendency toward overall airway narrowing.

Finally, the skeletal class did not show any statistically significant differences. Classes I and II did not differ from Class III in their association with either UPA1 or UPA2 (all p>0.2).

## Discussion

In this study, three distinct mandibular (Md-G1, Md-G2, and Md-G3) and three pharyngeal phenotypes (UPA1, UPA2, and UPA3) were identified through EFA and hierarchical clustering. These findings suggest that mandibular patterns characterized by vertical growth and transverse narrowing (Md-G1 and Md-G2) are predominantly associated with narrowed or collapsed airway configurations (UPA1 and UPA2), whereas the horizontally developed U-shaped mandible (Md-G3) tends to correspond to a wider and more stable airway (UPA3). These associations highlight the relevance of craniofacial morphology in determining upper airway variability.

The multivariate model confirmed the relationship between mandibular morphology and the airway phenotype. Compared with Md-G3, the Md-G1 phenotype showed a higher likelihood of belonging to UPA1 (RR=2.60; 95% CI: 0.94-7.23; p=0.066) and UPA2 (RR=2.96; 95% CI: 1.18-7.45; p=0.021), while Md-G2 was significantly associated with UPA1 (RR=2.81; 95% CI: 1.02-7.78; p=0.045). These associations suggest that mandibles with vertical and narrower configurations are more frequently linked to collapsed airway morphologies, whereas the Md-G3 phenotype more often corresponds to a wider airway. Although sex and age did not reach statistical significance in most comparisons, the increased probability of overall narrowing in the older groups indicates that age contributes to airway variability beyond skeletal morphology.

The present study provides morphometric evidence that mandibular and airway phenotypes may guide orthodontic diagnosis and treatment planning. These findings are consistent with previous reports showing that skeletal class II malocclusions are associated with reduced airway dimensions and a higher predisposition to collapse, particularly in the oropharyngeal region [24]. However, some earlier studies did not detect significant associations between sagittal relationships and airway volume, although they did report links with minimum CSAs [25]. Therefore, our results add nuance, indicating that mandibular morphology influences not only the sagittal but also the transverse airway dimensions. Other studies similarly support the role of the mandibular structure: retrognathic and class II morphologies have been linked to smaller pharyngeal spaces, while prognathic or class III mandibles tend to present larger volumes [5,6,26]. Moreover, mandibular shape also interacts with tongue posture and airway asymmetry, highlighting the complexity of the biomechanical determinants of airflow [7,27]. However, these suggested clinical applications should be interpreted as hypotheses to be validated, given that functional respiratory evaluation was not included in the present analysis.

The 3D characterization of mandibular and airway morphology using EFA allowed us to capture patterns that are often overlooked in linear cephalometric studies. For example, the Md-G2 group emerged as an intermediate form that cannot be distinguished by conventional angular classifications [5,11]. Similar findings have been described in studies linking open gonial angles to dolichofacial biotypes and broader morphologies to brachyfacial patterns [6-8]. The classification of airway phenotypes into UPA1, UPA2, and UPA3 likewise adds functional relevance as it enables the identification of narrowing patterns that are not reducible to simple upper/lower subdivisions [28]. Previous evidence has associated hyperdivergent growth with pharyngeal narrowing, comparable to UPA1, and hypodivergent forms with wider airways, consistent with UPA3.

Most of the available literature has relied on cephalometric methods, which separate linear and angular measures without accounting for 3D interdependence. This fragmentation prevents the integration of size, form, and spatial relationships. Decades ago, both Moyers and Bookstein emphasized the limitations of this reductionist view [29,30]. Although Procrustes-based geometric morphometrics advanced shape analysis by removing the effects of scale and position, it also eliminated biologically relevant variability. For this reason, our study analyzed raw landmark coordinates, preserving not only shape but also size and spatial positioning. Although this approach complicates strict shape comparisons, it allows recognition of two distinct but complementary phenomena: (1) positional effects of mandibular posture, which modify the hyoid position and suprahyoid muscle tension, thus influencing airway diameter, and (2) anatomical effects of mandibular base dimensions, where greater transverse development provides broader muscular support. The combination of these factors results in wider and more stable airway configurations.

CBCT imaging reinforced these observations, offering reliable 3D visualization of the bone and airway structures. Compared with cephalometry and MRI, CBCT provides superior detail of skeletal morphology, whereas functional techniques such as computational fluid dynamics (CFD) expand the understanding of airflow mechanics [31,32]. Together, these methods underline the clinical value of preserving dimensional and positional information in morphometric analyses, as performed in this study.

Adjustment of our models for age, sex, and skeletal class increased robustness and confirmed that mandibular morphology is an independent determinant of airway phenotype. This is consistent with evidence associating skeletal class II malocclusion with reduced airway volume and higher resistance, and class III malocclusion with larger airway dimensions [5,33]. Growth patterns also modulate this relationship; hyperdivergent patients tend to present narrower airways than hypodivergent ones [8,34]. Similarly, sex and facial type contribute to variability, with males and brachycephalic types generally showing wider airway dimensions [35,36]. These associations reinforce mandibular morphology as a structural determinant of airway phenotype, independent of demographic variables.

An important methodological distinction of this study was the decision to analyze raw landmark coordinates without applying Procrustes superimposition, instead recentering them at mandibular landmark 2 to eliminate translation. This approach preserves anatomical variability in size and spatial position, thereby maintaining functional information on mandibular and airway morphology that is often lost when only pure shape is considered. While Procrustes alignment facilitates shape comparisons across individuals, it constrains the interpretation of dimensional and positional variabilities that are clinically relevant for airway function. Similarly, EFA was chosen over PCA, as EFA is correlation-oriented and better suited to uncovering latent morphological structures, whereas PCA primarily maximizes variance without guaranteeing biological interpretability [22]. This methodological framework allowed us to detect intermediate mandibular phenotypes, such as Md-G2, and define airway patterns in a more functionally meaningful way. Nevertheless, these decisions also have limitations; preserving size and position complicates the separation of purely morphological effects from postural influences, which may affect interpretability. In addition, increasing the sample size or stratifying analyses between growing patients and adults could enhance factor stability and clarify whether developmental changes modify these mandibular-airway relationships.

Nevertheless, our findings should be cautiously interpreted. The associations were established on anatomical grounds without validation through functional outcomes such as polysomnography. Thus, while morphological patterns indicate a predisposition, their direct clinical impact on OSA remains hypothetical. Even so, the identification of mandibular phenotypes linked to airway narrowing has clinical implications for early recognition of patients at risk of obstruction and for guiding orthodontic and surgical interventions.

Clinical implications

Mandibles with vertical growth and reduced transverse dimensions appear as anatomical biomarkers of obstructive risk, particularly relevant for OSA, a condition with major systemic consequences [2,3,7,8]. This knowledge can inform orthodontic or orthognathic treatment planning and support therapeutic decisions, such as mandibular advancement or expansion [17]. Interventional studies have shown that functional appliances and advancement surgeries enlarge the airway and improve respiratory indices [36-38]. Conversely, mandibular setback procedures reduce the airway space and may worsen sleep-disordered breathing, although maxillary advancement may mitigate these effects [39-41]. These outcomes highlight the need to balance skeletal correction and airway preservation.

Limitations

Despite the use of 3D data and random sample selection, several limitations of this study must be acknowledged. This cross-sectional design prevents causal inference. For functional validation, neither sleep studies nor dynamic airflow assessments were performed, which limits clinical extrapolation and prevents determining whether the morphometric associations observed truly reflect OSA risk. The position of the hyoid bone, an important determinant of airway dimensions, was not included in the analysis [33,34]. In addition, other relevant variables such as tongue volume, body mass index, and respiratory habits were not assessed and could act as confounding factors [7,8,33]. The predominance of females in the sample may also represent a source of bias, as women are known to present smaller upper airway dimensions than men; consequently, our results could overestimate the frequency of reduced airway dimensions [36]. Finally, the sample was drawn from a single Mexican population, which may limit the generalizability of our findings to other ethnic or geographic groups. Even so, the preservation of size, form, and position in our morphometric analysis represents a methodological strength compared with prior cephalometric studies, enhancing the internal validity of the associations reported.

Future research directions

This study paves the way for future research using geometric morphometric methods based on Procrustes alignment (rotation, translation, and scaling), including techniques such as deformation analysis and configuration comparisons across diverse populations. Additionally, we propose that this functional morphology approach may serve as a screening tool for patients with a confirmed diagnosis of OSA to validate the anatomical phenotypes identified here as potential clinical risk predictors. Finally, longitudinal studies are needed to assess the evolution of mandibular and pharyngeal morphology during growth, as well as the impact of orthodontic or surgical interventions on the airway [7,8,13].

## Conclusions

Our study suggests a significant association between mandibular shape and the configuration of the pharyngeal airways. Phenotypes with vertical growth and narrow bases (Md-G1 and Md-G2) were associated with collapsed airway patterns (UPA1 and UPA2), whereas the horizontal and wide morphology (Md-G3) was related to wide and stable airways (UPA3). The multivariate model confirmed these associations after adjusting for age, sex, and skeletal class, highlighting that skeletal Class II is linked to sagittal collapse. These findings reinforce the clinical importance of considering 3D mandibular morphology as a structural marker of pharyngeal collapse risk, with relevant applications in orthodontics, orthognathic surgery, and sleep medicine. However, it must be noted that the cross-sectional design limits causal inferences.
